# Distance Surgical Mentorship for Ophthalmologists in Northern Peru

**DOI:** 10.15694/mep.2019.000045.1

**Published:** 2019-03-11

**Authors:** Amelia Geary, Sara Benavent, Edy Amador De La Cruz, Laura Wayman

**Affiliations:** 1 Orbis International; 2 Instituto Regional De Oftalmología Javier Servat Univazo; 3 Department of Ophthalmology and Visual Sciences at Vanderbilt University Medical Center

**Keywords:** Telementoring, Continuing Professional Development, Medical education, Surgical Skill Development

## Abstract

This article was migrated. The article was marked as recommended.

**Purpose:** To evaluate the quality of live distance surgical mentorship as an alternative way to provide continuing professional development (CPD) to practicing ophthalmologists. An activity which could be particularly beneficial to surgeons in remote locations where CPD is difficult to access.

**Methods:** Orbis paired ophthalmologists from the Regional Institute of Ophthalmology (IRO) in Trujillo, Peru and a senior ophthalmologist from Vanderbilt Eye Institute in Tennessee, USA (LW). One week in advance, the Peruvian surgeon sent the mentor patient information confidentially via Cybersight.org, Orbis’ telemedicine platform. The mentor reviewed the preoperative information to determine if the case was appropriate for remote guidance and formulated questions to help guide the educational experience. The mentor and mentee also consulted on specific learning objectives. The mentor observed live phacoemulsification surgery over the Internet using audio-visual equipment and Zoom desktop video conferencing software, allowing her to see through the operating microscope in real-time and have constant voice contact with the local surgeon. Post-mentorship a survey was administered to gauge acceptability of the CPD method, as well as their self-assessment on its impact on their skill development.

**Results:** Latency experienced was well within the suggested margin of acceptability and the video quality was broadcast-grade, allowing the mentor to clearly see the anatomy and instrument manipulation. Seven surgeons over four sessions performed twelve phacoemulsification surgeries in Peru, 91.67% of those 12 patients achieved postoperative best corrected visual acuity ≥6/18. Four surgeons completed the survey and 100% agreed or strongly agreed that their objectives had been met and that the CPD had increased their confidence and their surgical skills. The step in the procedure most commonly reported for improvement was nuclear cracking (75% of respondents), followed by hydrodissection, quadrant removal and wound closure (50% each).

**Conclusions:** Distance surgical mentorship in phacoemulsification is an acceptable form of CPD for consultant ophthalmologists. We describe a program with positive user feedback and experiences of improved confidence and microsurgical skill among participants.

## Introduction

Presently there are not enough ophthalmologists with competency in cataract surgical skills to meet the growing global need ( Resnikoff *et al.*, 2012 and Bourne *et al.,* 2017). This burden is unfairly distributed towards low and middle-income countries (LMICs), which severely lack ophthalmologists ( Naidoo *et al*., 2016). Among existing ophthalmologists, many are non-operative and of those that are, many have low surgical output ( Sommer *et al*., 2014). Additionally, studies show that visual outcomes of cataract surgery in many LMIC regions fail to meet the World Health Organization (WHO) recommended standards ( Lindfield *et al*., 2012). Many consultant ophthalmologists graduate from residency programs having not received adequate hands-on surgical training to ensure competency and confidence in cataract surgery, with some receiving none at all ( Thomas and Dogra, 2008 and Jiang, *et al*., 2016). This is coupled with the fact that many consultant ophthalmologists lack support networks and professional development opportunities ( IAPB, 2014).

The live distance surgical mentorship approach enables an expert ophthalmologist anywhere in the world to observe and coach local ophthalmologists on safe, high-quality surgical techniques by providing real-time video and audio feedback during cataract surgery. Studies have demonstrated distance mentorship as a reliable, efficient and cost-effective educational tool and model for mentorship ( Moore *et al*., 1996 and Challacombe *et al.,* 2006). However, technical problems, such as low video resolution and communication latency, may affect the quality of the mentorship delivery which may limit the benefits of this approach. In addition, ophthalmologists may not accept surgical mentorship delivered via distance learning, because they are uncomfortable with the technology or feel disconnected from their mentor. The purpose of this project is to evaluate the quality of live distance surgical mentorship as an alternative way for ophthalmologists in remote regions to obtain personalized CPD.

## Methods

Orbis evaluated the video-audio latency and video resolution of the live phacoemulsification cataract surgery between junior ophthalmologists at the Regional Institute of Ophthalmology (IRO) in Trujillo, Peru and a senior ophthalmologist (LW) at Vanderbilt Eye Institute in Tennessee, USA, using Zoom (San Jose, California) video conferencing software. Prior to the start of the project, the technical setting was established by the Orbis International IT department with a high-definition camera on the operating microscope, USB video capture card connected to a laptop, wired ethernet network connection, and an USB wireless lapel microphone. The quality of video and audio, and internet bandwidth performance and consistency were tested and found to be of excellent quality.

Enrollment for the remote surgical mentorship was voluntary, with consultants self-selecting to participate. The sessions were scheduled quarterly, with each session conducting 3 surgeries. Sessions were conducted in March, May, August and November 2018.

Before the live surgical mentorship sessions, the Peruvian surgeons sent the mentor patient information via Cybersight.org, including photos and lab work. All patients are counseled and consented not only on the surgery, but that the surgery will include telementorship. The surgeon completes a standardized form in Cybersight’s eConsultation, outlining the surgical plan and the mentor evaluates whether the case is appropriate for remote guidance. Additionally, mentor and mentees have one-on-one discussions where they identify goals for the remote mentorship session.

Surgeons performed twelve phacoemulsification surgeries in the Instituto Regional De Oftalmología operating room in Peru, while the mentor observed over the internet using audio-visual equipment and Zoom desktop video conferencing software, allowing her to see the surgeon’s view through the operating microscope in real-time while in constant voice contact with the local surgeon. All mentorship sessions were recorded to serve as a continued reference for mentees.

Following the surgery, mentees sent post-operative reports on every patient, including best corrected visual acuity (BCVA) of patients at one day post-operatively. A post-mentorship survey was administered to gauge acceptability of the CPD method, as well as their self-assessment on its impact on their skill development, which step of the procedure most benefited from this model of mentorship, and if they would like to participate in additional remote surgical mentorship sessions.

## Results

The quality of the audio and video was assessed as follows:

• Video latency from IRO to mentor: average of 226ms
• Audio latency from mentor back to IRO: average of 232ms
• Video resolution: 1280 x 720 pixels, at an average of 26 frames per second

Latency experienced is well within the suggestedmargin of acceptability ( Xu *et al*., 2014 and Fabrlzio, *et al*., 2000). Video quality was broadcast-grade, allowing the mentor to clearly see the anatomy and instrument manipulation and audio quality did not result in delays in communication.

In total, seven ophthalmologists opted to participate in the program, four male and three female surgeons. Of the seven, five were classified by their department head as having an intermediate skill level and two as having a basic skill level. The surgeons performed twelve surgeries in total. Patients ranged in age from 50-82 years, with the mean (standard deviation) age being 68 (9.8) years, and 7 (58%) of the patients were women. Fifty percent of patients (6) patients presented with one or more systemic or ocular comorbidity, most commonly diabetes (3) arterial hypertension (3) and suspected glaucoma (1). Post-operative, 91.76% of patients had a day one BCVA of ≥6/18, (range 6/7.5 -6/42, median 6/15) meeting the WHO criterion for quality cataract surgery (WHO, 2008).

Four of the seven participating surgeons completed the post-mentorship survey. Two females and two males. Three (75%) reported learning Phaco surgery on the job, and only one (25%) reported learning during residency. All agreed (75%) or strongly agreed (25%) that the Zoom platform was easy to use, and that the internet connection was stable. All strongly agreed (75%) or agreed (25%) that they were comfortable asking the mentor questions and that her responses were clear and informative. Further, 100% strongly agreed (75%) or agreed (25%) that the CPD had increased their confidence and their skills as a surgeon. The step in the procedure most commonly reported for improvement was nuclear cracking (75% of respondents), followed by hydrodissection, quadrant removal and wound closure (50% each). At least one respondent also reported improvements in each of the following steps: cortex removal, IOL insertion, nuclear sculpting, surgical prep and draping, temporal wound, and viscoelastic removal.

Surgeons unanimously agreed (75%) or strongly agreed (25%) that their objectives had been met, they would recommend telementorship to others (50% agreed and 50% strongly agreed) and that they would be interested in repeating this telementorship experience (50% agreed and 50% strongly agreed).

## Discussion

Telementoring in medicine is not an entirely new concept, and in other fields of medicine, particularly in laparoscopic procedures, has been shown to be successful ( Moore *et al.,* 1996). There is great potential for using telementoship to provide on-going continuing professional development for ophthalmologists practicing in remote settings or in countries where access to CPD is limited. Participants in telementorship report improved skills and at times, preference for the mode of teaching ( Campbell *et al.,* 2015 and Tang *et al*., 2017). In addition, there are potential cost benefits in limiting travel to mentors and mentees and to engage in in-person mentorship ( Means et al., 2013). Lastly, there is the potential benefit to the patient, with increased patient quality assurance.

The obvious barrier to telementorship is the requirement of audiovisual equipment and the need for reliable internet with appropriate bandwidth. IRO and Orbis invested in upgrading the internet connectivity of the operating room facilities, as well as the provision of the audio-visual equipment. The average cost for the AV equipment required ranges from $8,000 to $20,000USD depending on what is currently available at the facility. However, these are all fixed costs, once the investment is made, recurring costs are quite low with mentors volunteering their time, a one-time $5 user enrollment fee for Cybersight.org (currently borne by Orbis) and a pro-account subscription to Zoom costing $150/year.

Additionally, Rosser *et al*.,1999 applied telementoring in rural Ecuador using low bandwidth mobile telemedicine applications to support a mobile surgery program and were able to successfully perform a laparoscopic cholecystectomy mentored by the department of surgery at Yale University School of Medicine. Demonstrating that it is possible to offer telementorship in locations without the use of sophisticated technology or high-speed internet.

Thus far, the data generated from this project, is from a small sample size (N=7). However, IRO has currently adopted this mode of teaching and the twining between Vanderbilt and IRO is entering its second year. Additionally, Orbis is currently investing in upgrading the infrastructure at partner hospitals in Bolivia, Mongolia, Ghana and Zambia to expand the telementorship portfolio. The successful implementation at these various locations representing a diversity of patient populations, surgical skills, infrastructure and access, will generate additional data on this method of mentorship.

## Conclusion

The project demonstrated that distance surgical mentorship in phacoemulsification is an acceptable form of obtaining CPD for consultant ophthalmologists. So far user feedback is positive, and participants report improved confidence and technical skill. Surgical cases performed under this mentorship were successful, with good patient outcomes. Telementorship can help overcome access and cost barriers for ophthalmologists globally trying to obtain (or maintain) CPD.

## Take Home Messages

• Telementorship in phacoemulsification surgery is an acceptable form of CPD for consultant ophthalmologists.
• Surgeries performed under telementorship had good patient outcomes.
• Mentees report improved confidence and skills as a result of telementorship.
• Telementorship can provide access to CPD for ophthalmologists working in remote areas with limited support and professional development opportunities.

## Notes On Contributors

Amelia Geary is Director of Program Development & Quality for Orbis International. She studied International Develop and Infrastructure Development at Columbia University in New York City, USA. Under her portfolio, she leads the training and innovation team, developing new training modalities and approaches for ophthalmic medical education. Her focus has been largely blended learning, distance learning and simulation training.

Sara Benavent is the Senior Program Manager, Latin American and the Caribbean region, for Orbis International. With a master’s degree in International Development and Population Sciences from the University of Brussels, she has broad experience in socio economic research, strategy and policy design and field program management. She has successfully led initiatives in capacity building in the public sector.

Edy Amador De La Cruz, MD obtained his degree in medicine at the Faculty of Medicine of the National University of Trujillo (UNT). He is currently a consultant ophthalmologist at the Instituto Regional De Oftalmología Javier Servat Univazo.

Laura Wayman, MD is vice chair for education in the Department of Ophthalmology and Visual Sciences at Vanderbilt University Medical Center. She has served on the American Academy of Ophthalmology Committee for Resident Education and was president of the Association of University Professors in Ophthalmology Program Directors Council. She currently serves on the Accreditation Council for Graduate Medical Education Ophthalmology Residency Review Committee.

## References

[ref1] Bourne R. R. A. Flaxman S. R. Braithwaite T. Cicinelli M. V. (2017) Magnitude, temporal trends, and projections of the global prevalence of blindness and distance and near vision impairment: a systematic review and meta-analysis. The Lancet Global Health. 5(9), pp.e888–e897. 10.1016/S2214-109X(17)30293-0 28779882

[ref2] Campbell J. P. Swan R. Jonas K. Ostmo S. (2015) Implementation and evaluation of a tele-education system for the diagnosis of ophthalmic disease by international trainees. AMIA Annual Symposium Proceedings Archinve. 2015, pp.366–375. Available at: https://www.ncbi.nlm.nih.gov/pmc/articles/PMC4765571/ PMC4765571 26958168

[ref3] Challacombe B. Kandaswamy R. Dasgupta P. and Mamode N. (2006) Telementoring Facilitates Independent Hand-Assisted Laparoscopic Living Donor Nephrectomy. Journal of Urology. 175(1), pp.237–237. 10.1016/s0022-5347(05)00085-6 15848474

[ref4] Fabrlzio M. D. Lee B. R. Chan D. Y. Stoianovici D. (2000) Effect of Time Delay on Surgical Performance During Telesurgical Manipulation. Journal of Endourology. 14(2), pp.133–138. 10.1089/end.2000.14.133 10772504

[ref5] IAPB (2014). ADDRESSING THE EYE HEALTH WORKFORCE CRISIS IN SUB-SAHARAN AFRICA: BUSINESS AS USUAL IS NOT AN OPTION.- Available at: https://www.iapb.org/wp-content/uploads/Addressing-the-Eye-Health-Workforce-Crisis-in-Sub-Saharan-Africa.pdf( Accessed: February 28, 2019).

[ref6] Jiang Y. Luo L. Congdon N. Wang S. (2016) Who will be wielding the lancet for Chinas patients in the future? The Lancet. 388(10054), pp.1952–1954. 10.1016/s0140-6736(16)31792-5 27751402

[ref7] Lindfield R. Ngounou F. Vishwanath K. and Khanna R. (2012) The challenges in improving outcome of cataract surgery in low and middle income countries. Indian Journal of Ophthalmology. 60(5), p.464. 10.4103/0301-4738.100552 22944761 PMC3491277

[ref8] Means B. Toyama Y. Murphy R. and Baki M. (2013) The effectiveness of online and blended learning: A meta-analysis of the empirical literature. Teachers College Record. 115, pp.1–47. Available at: https://www.sri.com/sites/default/files/publications/effectiveness_of_online_and_blended_learning.pdf

[ref9] Moore R. G. Adams J. B. Partin A. W. Docimo S. G. (1996) Telementoring of laparoscopic procedures. Surgical Endoscopy. 10(2), pp.107–110. 10.1007/bf00188353 8932609

[ref10] Naidoo K. S. Leasher J. Bourne R. R. Flaxman S. R. (2016) Global Vision Impairment and Blindness Due to Uncorrected Refractive Error, 1990-2010. Optometry and Vision Science. 93(3), pp.227–234. 10.1097/opx.0000000000000796 26905537

[ref11] Resnikoff S. Felch W. Gauthier T.-M. and Spivey B. (2012) The number of ophthalmologists in practice and training worldwide: a growing gap despite more than 200 000 practitioners. British Journal of Ophthalmology. 96(6), pp.783–787. 10.1136/bjophthalmol-2011-301378 22452836

[ref12] Rosser J. C. Bell R. L. Harnett B. Rodas E. (1999) Use of mobile low-bandwith telemedical techniques for extreme telemedicine applications. Journal of the American College of Surgeons. 189(4), pp.397–404. 10.1016/s1072-7515(99)00185-4 10509466

[ref13] Sommer A. Taylor H. R. Ravilla T. D. West S. (2014) Challenges of Ophthalmic Care in the Developing World. JAMA Ophthalmology. 132(5), p.640. 10.1001/jamaophthalmol.2014.84 24604415 PMC4063878

[ref14] Tang F. Chen C. Zhu Y. Zuo C. (2017) Comparison between flipped classroom and lecture-based classroom in ophthalmology clerkship. Medical Education Online. 22(1), p.1395679. 10.1080/10872981.2017.1395679 29096591 PMC5678346

[ref15] Thomas R. and Dogra M. (2008) An evaluation of medical college departments of ophthalmology in India and change following provision of modern instrumentation and training. Indian Journal of Ophthalmology. 56(1), p.9. 10.4103/0301-4738.37589 18158398 PMC2636055

[ref16] World Health Organization .1998. Informal Consultation on Analysis of Blindness Prevention Outcomes. WHO/PBL/98.68, Geneva, WHO.

[ref17] Xu S. Perez M. Yang K. Perrenot C. (2014) Determination of the latency effects on surgical performance and the acceptable latency levels in telesurgery using the dV-Trainer® simulator. Surgical Endoscopy. 28(9), pp.2569–2576. 10.1007/s00464-014-3504-z 24671353

