# Supplementary material for: Distance Surgical Mentorship for Ophthalmologists in Northern Peru
Source: MedEdPublish (2016). 2019 Mar 11;8:45. [Version 1] doi: 10.15694/mep.2019.000045.1 (PMC10712597; doi:10.15694/mep.2019.000045.1)
Supplement: Peer review history

# Reviewer report: Reviewer response for version 1

**DOI:** 10.21956/mep.19800.r29785

**Published:** 2019-07-12

**Authors:** Trevor Gibbs

**Affiliations:** 1 AMEE

This review has been migrated. The reviewer awarded 4 stars out of 5 A very practical and well described paper that I think adds greatly to our thinking in the area of distant practical applications. I am not too worried that the numbers are small, this can be accepted.I agree that there are knowledge / information gaps within the paper but these can be easily resolved by those wishing more details.

Reviewer Expertise:

NA

No decision status is available

# Reviewer report: Reviewer response for version 1

**DOI:** 10.21956/mep.19800.r29784

**Published:** 2019-06-30

**Authors:** Ken Masters

**Affiliations:** 1 Sultan Qaboos University

This review has been migrated. The reviewer awarded 3 stars out of 5 An interesting paper dealing with distance surgical mentorship for ophthalmologists in Northern Peru. The paper clearly explains the scope of the problem: too few trained surgical ophthalmologists worldwide, and even more so in low-income countries.The study is rather small, but does have value for other researchers.Some areas that should be addressed in the paper:• Although the paper adequately discusses the problems worldwide and in low-income countries, it does not give any contextual information about Peru, where the study is set. For the reader to have an appreciation of the value of such an exercise, it is crucial for this contextual information to be given upfront, in the Introduction.• No information is given about Orbis, and the authors too quickly launch in the findings. Is Orbis an organisation? A company? A government funded project? What does it do? (I understand the authors would be loath to appear to be advertising, but some contextual information is needed).• In the Methods, the tense changes from past to present and then back to past. The authors really should select one tense (I suggest past) and then stick to it.• There needs to be more consistency in the reporting of the statistics. Sometimes we see raw numbers and then percentages, sometimes this is revered, and sometimes we see only one without the other. The recommended format is raw number followed by percentage (to at least one decimal place) in brackets.• It would be useful if the researchers could supply a copy of the survey questionnaire.• With reference to Orbis above, I strongly urge the authors to mention in the potential conflict of interests that the lead and second author are at Orbis, the company that funded the project, and whose equipment was used in the project. I realise that they have given this information in their Notes on Contributors, but it needs to be stated in the potential conflict of interests also.So, the study appears to have had value, and is a useful start in the area. The authors, however, do need to strengthen their paper somewhat. I look forward to seeing a revised version of the paper in which the weaknesses are addressed.

Reviewer Expertise:

NA

No decision status is available

# Reviewer report: Reviewer response for version 1

**DOI:** 10.21956/mep.19800.r29783

**Published:** 2019-03-27

**Authors:** Helena Filipe

**Affiliations:** 1 Hospital of the Armed Forces/PL-EMGFA

This review has been migrated. The reviewer awarded 5 stars out of 5 The work of Geary and associates on the challenges of mentorship using live distance technology to outreach geographically remote ophthalmic surgeons within CPD is very useful and exciting.The Authors adequately inform their research background on evidence underlining the core role of mentorship in the process of developing competencies in ophthalmic surgery. They underline a special focus on those surgeons in remote regions and the global constraint of ophthalmologists and resources and adequately define their research goal.Methods are thoroughly defined to the point of replication. The section of results is clear and provides the ground for the following section of discussion. The process through which the chosen teaching methodology works is discussed with an emphasis on its feasible and well accepted application to teach and learn within CPD in low resources and remote settings. The conclusion is appropriate and the take home messages are well defined and integrate well with the results.I hope to see a next paper, regarding the application of this same model, replicated in other regions of the globe also in need of twinning, using telementorship and thus overcoming human and financial resources constraints. This is an excellent idea that was brilliantly adapted into practice with promising results. I would love to try this method while teaching in difficult and geographically remote circumstances.

Reviewer Expertise:

NA

No decision status is available
